# Ambulance use in Pakistan: an analysis of surveillance data from emergency departments in Pakistan

**DOI:** 10.1186/1471-227X-15-S2-S9

**Published:** 2015-12-11

**Authors:** Nukhba Zia, Hira Shahzad, Syed Muhammad Baqir, Shahab Shaukat, Haris Ahmad, Courtland Robinson, Adnan A Hyder, Junaid Abdul Razzak

**Affiliations:** 1 Department of Emergency Medicine, Aga Khan University, Karachi, Pakistan; 2 Johns Hopkins International Injury Research Unit, Department of International Health, Johns Hopkins Bloomberg School of Public Health, Baltimore, Maryland, USA; 3 Department of Emergency Medicine, Johns Hopkins School of Medicine, Baltimore, Maryland, USA; 4 The author was affiliated with the Department of Emergency Medicine, Aga Khan University, Karachi, Pakistan at the time when study was conducted

**Keywords:** Ambulance use, emergency departments, Pakistan

## Abstract

**Background:**

The utilization of ambulances in low- and middle-income countries is limited. The aim of this study was to ascertain frequency of ambulance use and characteristics of patients brought into emergency departments (EDs) through ambulance and non-ambulance modes of transportation.

**Methods:**

The Pakistan National Emergency Departments Surveillance (Pak-NEDS) was a pilot active surveillance conducted in seven major tertiary-care EDs in six main cities of Pakistan between November 2010 and March 2011. Univariate and multivariate logistic regression was performed to investigate the factors associated with ambulance use.

**Results:**

Out of 274,436 patients enrolled in Pak-NEDS, the mode of arrival to the ED was documented for 94. 9% (n = 260,378) patients, of which 4.1% (n = 10,546) came to EDs via ambulances. The mean age of patients in the ambulance group was significantly higher compared to the mean age of the non-ambulance group (38 ± 18.4 years versus 32.8 ± 14.9 years, p-value < 0.001). The most common presenting complaint in the ambulance group was head injury (12%) while among non-ambulance users it was fever (12%). Patients of all age groups were less likely to use an ambulance compared to those >45 years of age (p-value < 0.001) adjusted for gender, cities, hospital type, presenting complaint group and disposition. The adjusted odds ratio of utilizing ambulances for those with injuries was 3.5 times higher than those with non-injury complaints (p-value < 0.001). Patients brought to the ED by ambulance were 7.2 times more likely to die in the ED than non-ambulance patients after adjustment for other variables in the model.

**Conclusion:**

Utilization of ambulances is very low in Pakistan. Ambulance use was found to be more among the elderly and those presenting with injuries. Patients presenting via ambulances were more likely to die in the ED.

## Background

The World Health Organization (WHO) emphasizes the importance of emergency medical services (EMS) systems which are usually the first point of contact with healthcare systems for acute conditions like injuries, chest pain or acute presentation of chronic conditions like diabetic coma in diabetic patients [1]. Many high-income countries (HICs) have well-developed pre-hospital emergency systems which employ modern patient monitoring equipment and have paramedical staff trained to provide pre-hospital care in accordance with the patient's condition [2]. Since numerous acute conditions such as chest pain and hemorrhage are time-sensitive, established pre-hospital services play a crucial role in overall patient outcomes [3].

The situation is different in many low-and middle-income countries (LMICs) where there is lack of ambulance services and many patients present to acute healthcare facilities on their own. It is important to note that more than one-third of all deaths in LMICs are preventable with early intervention during the pre-hospital phase [4]. Up to 90% of injury-related mortality occurs in LMICs and in the presence of an adequate EMS system; this burden can be reduced by 45% [1,5]. However, due to lack of funding and trained personnel, emergency services in LMICs are a low priority and are often limited to providing basic transportation facilities without efficient triage services. There is also a general lack of public trust in ambulance services in LMICs [6]. The lack of pre-hospital care services in Pakistan has been a cause of great concern over the years [4,6-9].

The aim of this study was to determine the frequency of ambulance use by patients coming to the major emergency departments (EDs) of Pakistan. It will also compare the characteristics of patients coming by ambulances and those coming by other modes of transportation, such as public and private vehicles or walk-in patients to the EDs.

## Methods

### Study setting

The Pakistan National Emergency Departments Surveillance (Pak-NEDS) was a pilot active surveillance conducted in seven major tertiary care emergency departments in six main cities of Pakistan between November 2010 and March 2011. The EDs included the Aga Khan University (Karachi), Jinnah Post-Graduate Medical Center (Karachi), Mayo Hospital (Lahore), Sandeman Provincial Hospital (Quetta), Lady Reading Hospital (Peshawar), Benazir Bhutto Hospital (Rawalpindi), and Shifa International Hospital (Islamabad). Five of the participating hospitals were public hospitals and two were private hospitals. All the hospitals are tertiary care teaching hospitals and one is a referral center. Ethical approval was obtained from all participating hospitals.

There are several ambulance services operating in various regions of the country which include philanthropic organizations such as the Edhi Foundation (nationwide), and Chippa Welfare Association (Karachi) [10,11]. The Aman Foundation is another non-profit organization in Karachi which deals with healthcare, education and skills, and nutrition for underprivileged [12]. A few EMS services arose due to efforts of provincial governments such as Rescue 1122 [13]. However, these ambulance services currently work in Pakistan at the local level and are not part of an integrated emergency care system. Pak-NEDS did not collect data from any of the above mentioned ambulance services.

### Study procedure

Data collectors were specifically hired and trained for Pak-NEDS. They worked in three shifts providing 24/7 coverage. Data was collected from patients or the next of kin and ED records. A one-page standardized tool was developed based on an ambulatory care survey tool developed by the Centers for Disease Control and Prevention, USA and on previous surveillance work done in Pakistan [14,15]. The tool gathered information related to patient demographics, presenting complaints, treatment and management provided in the ED, provisional diagnosis and disposition from the ED. For this study, we looked at the mode of arrival to the ED of all the patients enrolled in Pak-NEDS. The mode of ED arrival was categorized into two groups; ambulance and non-ambulance. Non-ambulance group comprised of patients who presented to the ED through other means of transport like private vehicle.

### Data management

Data was entered at AKU using EpiInfo version 3.3.2, and SPSS version 19 was used for analysis [16,17]. For the purpose of analysis, six age categories were developed; less than 5 years, 5-12 years, 13-18 years, 19-25 years, 26-45 years, and more than 45 years. Pak-NEDS recorded up to three presenting complaints. In this analysis, presenting complaint is used as a multiple response variable. All presenting complaints were categorized into two major categories, injuries and non-injury.

Injuries included unintentional and intentional injuries. The types of injuries recorded were falls, burns, drowning, poisoning, road traffic injuries and firearm injuries. Non-injury included general presenting complaints like fever, fatigue, weakness, swelling and complaints based on the body organ involved; for example, chest pain was grouped under cardiovascular system, rectal bleeding as part of gastrointestinal system. Other systems in this category were respiratory, central nervous system, musculoskeletal, head and neck, and uro-gynecology. Cities were grouped together by geographical location of participating hospitals: Aga Khan University and Jinnah Post-graduate Medical Center in Karachi; Mayo Hospital in Lahore; Benazir Bhutto Hospital and Shifa International Hospital in Rawalpindi/Islamabad; Lady Reading Hospital in Peshawar; and Sandeman Provincial Hospital in Quetta.

### Data analysis

Comparison of patient factors was done between ambulance and non-ambulance groups using Pearson's Chi-squared test for categorical variables and independent sample t-test for continuous variables with level of significance set at 0.05. We also looked at the use of ambulance as an outcome variable. Univariate and multivariate logistic regression was carried out to look at the factors associated with ambulance use. A multivariate model was developed with independent variables including gender, age group, city, hospital type, presenting complaint and disposition.

## Results

Out of 274,436 patients enrolled into Pak-NEDS, the mode of arrival to the ED was documented for 94.9% (n = 260,378). Out of these, 4.1% (n = 10,546) were brought to the ED via ambulances; the rest of the patients (n = 249,832, 95.9%) were brought by means other than ambulance. This means that the overall use of ambulance services by patients coming to the major EDs in Pakistan for acute care is 1 in 25 patients.

Comparison of demographic characteristics of patients between the ambulance and non-ambulance group is given in Table 1. In the ambulance group, the proportion of males was 63.4% (n = 6578), while the proportion of males in the non-ambulance group was 60.7% (n = 150,085). The mean age of patients in the ambulance group (38 ± 18.4 years) was significantly higher (p-value < 0.001) compared to the mean age of the non-ambulance group (32.8 ± 14.9 years). The proportion of patients brought to public versus private hospitals in both ambulance and non-ambulance groups was similar (93.7% vs. 93.9%, p-value 0.347).

**Table 1:** Comparison of demographics characteristics and outcome of patients between ambulance and non-ambulance groups

Patient characteristics	Ambulance (n = 10546, 4.1%)	Non-ambulance (n = 249832, 95.9%)	p-value	Total (n = 260,378)
			
	n (%)	n (%)		n(%)
**Age in years (mean ± SD)**	38 ± 18.4	32.8 ± 14.9	< 0.001	33.1 ± 15.3

**Gender (n = 257,684)**			< 0.001	
Male	6578 (63.4)	150,085 (60.7)		156,663 (60.8)
Female	3795 (36.6)	97,226 (39.3)		101,021 (39.2)

**Age groups (n = 250,034)**				
< 5 years	122 (1.2)	3793 (1.6)	< 0.001	3915 (1.6)
5 - 12 years	335 (3.3)	10,057 (4.2)		10,392 (4.2)
13 - 18 years	747 (7.4)	22,053 (9.2)		22,800 (9.1)
19 - 25 years	1761 (17.5)	52,490 (21.9)		54,251 (21.7)
26 - 45 years	4224 (41.9)	109,410 (45.6)		113,634 (45.4)
>45 years	2896 (28.7)	42,146 (17.6)		45,042 (18)

**Hospital type (n = 260,378)**			0.347	
Public	9883 (93.7)	234,683 (93.9)		244,566 (93.9)
Private	663 (6.3)	15,149 (6.1)		15,812 (6.1)

Presenting complaint group* (n = 230,163)			< 0.001	
Non-injury	7262 (82.8)	247,476 (111.8)		254,738 (110.7)
Injuries	5187 (59.1)	50,589 (22.9)		55,776 (24.2)

**Disposition (n = 185,370)**			< 0.001	
Discharged from ED	5025 (59)	143,891 (81.4)		148,916 (80.3)
Admitted	2891 (33.6)	26410 (14.9)		29,301 (15.8)
Death in ED	341 (4)	1468 (0.8)		1809 (1.0)
Others**	266 (3.1)	5078 (2.9)		5344 (2.9)

*multiple response variable therefore the total is not be 100%

**includes referred patients, left without being seen, left against medical advice

Overall injuries were the presenting complaint in 24.4% (n = 55,776) of all ED visits. Among ambulance users, the proportion of patients with injuries was 59.1% (n = 5187). The analysis shows that in Karachi, 9.4% of ED patients arrived by ambulance versus only 3.4% in Lahore, 2.8% in Peshawar, 2.7% in Quetta and 1.0% in Rawalpindi/Islamabad (Table 2).

**Table 2:** Use of ambulance by emergency department patients in different cities of Pakistan (n = 260,378)

Cities*	Ambulance group	Non-ambulance group	Total
	
	n (%**)	n (%**)	n(%***)
Karachi	5807 (9.4)	55,930 (90.5)	61,737 (23.7)
Lahore	1589 (3.6)	43,081 (96.4)	44,670 (17.2)
Peshawar	1578 (2.8)	53,319 (97.1)	54,897 (21.1)
Quetta	912 (2.7)	32,354 (97.3)	33,266 (12.8)
Rawalpindi/Islamabad	660 (1.0)	65,148 (99.0)	65,808 (25.3)

* Cities variable was created based on the geographical location of participating hospitals; Aga Khan University and Jinnah Post-graduate Medical Center in Karachi; Mayo Hospital in Lahore, Benazir Bhutto Hospital and Shifa International Hospital in Rawalpindi/Islamabad; Lady Reading Hospital in Peshawar and Sandeman Provincial Hospital in Quetta

**Percentage based on row total

***Percentage based on column total

The most common presenting complaint in patients using ambulance services was head injury while among non-ambulance users it was fever. Table 3 lists the top ten presenting complaints in both the ambulance and non-ambulance group. Among different age groups within the ambulance use group, injury was the most common reason for coming to the ED for patients in the under 5 years to 26-45 years age group; however, patients above 45 years of age presented to the ED due to non-injury complaints. Among those arriving by ambulances, the proportion being admitted was more than two times compared to those in the non-ambulance group (33.6% vs. 14.9%). About 4% of patients in the ambulance group died in the ED (Table 1).

**Table 3:** Top 10 presenting complaints in ambulance and non-ambulance group

Ambulance*	Non-ambulance**
Head injury (12%)	Fever (12%)
Lower extremity injury (11%)	Abdominal pain (9%)
Loss of consciousness (7%)	Vomiting (7%)
Chest pain (6%)	Chest pain (7%)
Shortness of breath (6%)	Headache (5%)
Fever (5%)	Cough (4%)
Upper limb injuries (5%)	Lower extremity injuries (4%)
Abdominal pain (5%)	Shortness of breath (4%)
Face injuries (4%)	Head injury (3%)
Vomiting (4%)	Upper limb injuries (3%)

* constitutes 65% of all presenting complaints in non-ambulance group

**constitutes 59% of all presenting complaints in non-ambulance group

To examine the factors related to use of ambulance, logistic regression was performed on data available for 154,200 (56.2%) cases (Table 4). Patients of age groups <45 years were less likely to be transported by ambulance compared to more than 45 years age group (p-value < 0.001) adjusted for gender, cities, hospital type, presenting complaint group and disposition. The association of ambulance use with gender was not statistically significant in the model after adjusting for other independent variables in the model. The adjusted odds ratio of utilizing ambulances for those with injuries was 3.5 times higher than those presenting with non-injury complaints (p-value < 0.001).

**Table 4:** Logistic regression of factors associated with ambulance use

Patient characteristics	Univariate regression			Multivariate regression		
	
	Unadjusted ORs	95% Confidence interval	p-value	Adjusted ORs*	95% Confidence interval	p-value
**Gender**						
Female	**REF**			**REF**		
Male	1.12	1.1, 1.2	< 0.001	1.0	1.0, 1.1	0.5

**Age groups**						
> 45 years	**REF**			**REF**		
< 5 years	0.5	0.4, 0.6	< 0.001	0.3	0.3, 0.4	< 0.001
5 - 12 years	0.5	0.4, 0.5	< 0.001	0.3	0.3, 0.4	< 0.001
13 - 18 years	0.5	0.4, 0.5	< 0.001	0.4	0.4, 0.5	< 0.001
19 - 25 years	0.5	0.4, 0.5	< 0.001	0.4	0.4, 0.5	< 0.001
26 - 45 years	0.6	0.5, 0.6	< 0.001	0.5	0.4, 0.5	< 0.001

**Cities**						
Quetta	**REF**			**REF**		
Karachi	3.7	3.4, 4.0	< 0.001	3.6	3.2, 4.1	< 0.001
Lahore	1.3	1.2, 1.4	< 0.001	1.6	1.4, 1.8	< 0.001
Rawalpind/Islamabad	0.4	0.3, 0.4	< 0.001	0.3	0.3, 0.4	< 0.001
Peshawar	1.1	1.0, 1.1	0.25	0.6	0.5, 0.7	< 0.001

**Hospital type**						
Private	**REF**			**REF**		
Public	0.9	0.9, 1.0	0.35	2.3	2.1, 2.6	< 0.001

**Presenting complaint**						
Non-injury	**REF**			**REF**		
Injuries	3.2	3.0, 3.3	< 0.001	3.5	3.3, 3.7	< 0.001

**Disposition**						
Discharged from ED	**REF**			**REF**		
Admitted	3.1	3.0, 3.3	< 0.001	3.1	2.9, 3.3	< 0.001
Death in ED	6.7	5.9, 7.5	< 0.001	7.2	6.2, 8.4	< 0.001
Others**	1.5	1.3, 1.7	< 0.001	1.4	1.2, 1.6	< 0.001

OR = odds ratio

*Model constant -4.3

**includes referred patients, left without being seen, left against medical advice

The adjusted odds of admission among patients in the ambulance group were 3.0 times higher compared to patients in the non-ambulance group (p-value < 0.001). Patients brought to the ED by ambulance were 7.3 times more likely to die in the ED than non-ambulance patients, after adjusting for gender, age groups, cities, hospital type and presenting complaint group (p-value < 0.001).

## Discussion

This study shows that only 4.1% of the patients coming to the major EDs in Pakistan use ambulance services. There are no global standards for appropriate utilization rate for ambulances. Many factors are likely to play a significant role in the utilization rate including availability of ambulances, cost of the service, differences in the disease burden and severity of illnesses, age distribution of population, geographic spread and availability of alternate methods of seeking care. Nevertheless, the utilization rate from Pakistan is lower then reported from high income countries which have reported percentage of ambulance use between 14.2% and 30% for patients coming to the emergency department [18-20]. An even higher percentage of 67.3% was reported in a recent study from India [21]. A study done over 15 years ago in the city of Karachi, Pakistan reported ambulance use of 16%, though that study only looked at patients admitted to the hospital where the acuity, and therefore ambulance utilization, was expected to be higher than all who came to the emergency department [22]. This low use of ambulance in Pakistan has previously been attributed to poorly equipped transportation facilities, lack of proficient staff, and lack of pre-hospital care, all of which breed a lack of trust in the EMS system [4]. There is lack of trust in EMS by the public which augments uncertainty about the adequacy of EMS in Pakistan to provide pre-hospital care [6]. Pak-NEDS shows that patients coming to the ED were likely to be elderly patients, those with injuries especially head injuries, and those who were likely to die in the ED. We did not find significant association between ambulance use and gender after controlling for other variables in the model.

This study shows that patients more than 45 years of age are more likely to come to ED via ambulance and most of the older patients present due to non-injury complaints. This is consistent with previous studies on ambulance utilization [23-25]. This could be because elderly patients have chronic diseases like diabetes, cardiovascular disease and acute deterioration of a chronic illness, which results in frequent ED visits. Also due to functional restraints, it is likely that ambulances are used to transport older patients to a nearby health facility for acute care [23]. In comparison to older patients, injury was the common reason for transporting younger patients to ED via ambulance. This might be due to quick response of ambulances in urban centers to such incidents.

A higher proportion of patients brought into the ED via ambulances died in the ED or were admitted to the hospital, compared to those who used other modes of transportation to the ED. Most of the deaths in the ambulance group were seen in patients presenting with chest pain or shortness of breath. This indicates the need for the establishment of pre-hospital emergency services, which encompass the provision of patient monitoring devices, basic and advanced life support, and trained paramedics in accordance with the availability of resources [1]. A study done in Canada reported that older patients with myocardial infarction who used ambulances were sicker when compared with their counterparts who did not use ambulances to come to the hospital [26]. Triage and pre-hospital care by paramedic staff remains an important constituent of emergency care. This is especially true for patients with time-sensitive conditions like myocardial infarction, stroke (hemorrhage/ischemia), sepsis, cardiopulmonary arrest and trauma, where prompt identification and treatment results in markedly improved patient survival and outcomes [27-31].

## Limitations

There are several limitations in this analysis. There was missing data in Pak-NEDS related to ambulance use. As a result, logistic regression was done on 56% of the patient for whom data related to all variables was available. The data lacked in information related to type of ambulance (transport vehicle, basic life support or advance life support vehicle) used for transportation of the patient, ambulance response time, transportation time and interventions done during the pre-hospital phase, if any, to the patients who came through ambulances. Our study recorded information related to different types of presenting complaints; for example, chest pain, injuries, and stroke. However, it lacks information on severity of these time-sensitive conditions. This hampers analysis related to disease severity and outcome. We did not have follow-up information on the patients to determine outcomes such as 30-day mortality or length of hospital stay, which would help determine the effectiveness of care provided in the emergency department as well as in the ambulance, if any. This study also lacks population level estimates related to ambulance use and hospital catchment area.

## Conclusion

This study shows that the use of ambulance services in Pakistan remains quite low overall. Patients older than 45 years of age and those who have injuries are more likely to be transported via ambulance. Patients coming to ED by ambulance have higher likelihood of death in the ED or admission to the hospital for further care. We propose that increasing utilization of a pre-hospital emergency care system integrated with overall healthcare system could potentially reduce mortality and improve outcomes. This needs further studies to see association between ambulance and better outcome.

## Competing interests

The authors declare that they have no competing interests.

## Authors' contributions

NZ was involved in the analysis and manuscript writing. HS, SS and HA wrote the first draft. SMB, CR, AAH and JAR provided critical review of the draft. AAH and JAR conceptualized Pak-NEDS and provided supervision during development of manuscript. All the authors approved the final manuscript except SS who passed away during the manuscript finalization phase.
